# Supplementary material for: Momentum-space signatures of Berry flux monopoles in the Weyl semimetal TaAs
Source: Nat Commun. 2021 Jun 15;12:3650. doi: 10.1038/s41467-021-23727-3 (PMC8206138; doi:10.1038/s41467-021-23727-3)
Supplement: Supplementary file 1 — Supplementary Information [file 41467_2021_23727_MOESM1_ESM.pdf]

Supplementary Information: Momentum-space signatures of Berry flux monopoles in the Weyl semimetal TaAs

M. Ünzelmann,* H. Bentmann,* T. Figgemeier,* B. Geldiyev, and F. Reinert

Experimentelle Physik VII and Würzburg-Dresden Cluster of Excellence ct.qmat, Universität Würzburg, Am Hubland, D-97074 Würzburg, Germany

P. Eck,* R. Thomale, and G. Sangiovanni

Theoretische Physik I, Universität Würzburg, Am Hubland, D-97074 Würzburg, Germany

J. N. Neu and T. Siegrist

Department of Chemical and Biomedical Engineering, FAMU-FSU College of Engineering Tallahassee, FL 32310, USA and National High Magnetic Field Laboratory, Tallahassee, FL 32310, USA

F. Diekmann, S. Rohlf, J. Buck, and M. Kalläne

Institut für Experimentelle und Angewandte Physik, Christian-Albrechts-Universität zu Kiel, D-24098 Kiel, Germany and Ruprecht Haensel Laboratory, Kiel University and DESY, Germany

M. Hoesch

Deutsches Elektronen-Synchrotron DESY, D-22607 Hamburg, Germany

K. Rossnagel

Institut für Experimentelle und Angewandte Physik, Christian-Albrechts-Universität zu Kiel, D-24098 Kiel, Germany Ruprecht Haensel Laboratory, Kiel University and DESY, Germany and Deutsches Elektronen-Synchrotron DESY, D-22607 Hamburg, Germany

D. Di Sante

Theoretische Physik I, Universität Würzburg,

Am Hubland, D-97074 Würzburg, Germany
Department of Physics and Astronomy,
University of Bologna, 40127 Bologna, Italy and
Center for Computational Quantum Physics, Flatiron Institute,
162 5th Avenue, New York, New York 10010, USA

(Dated: April 26, 2021)

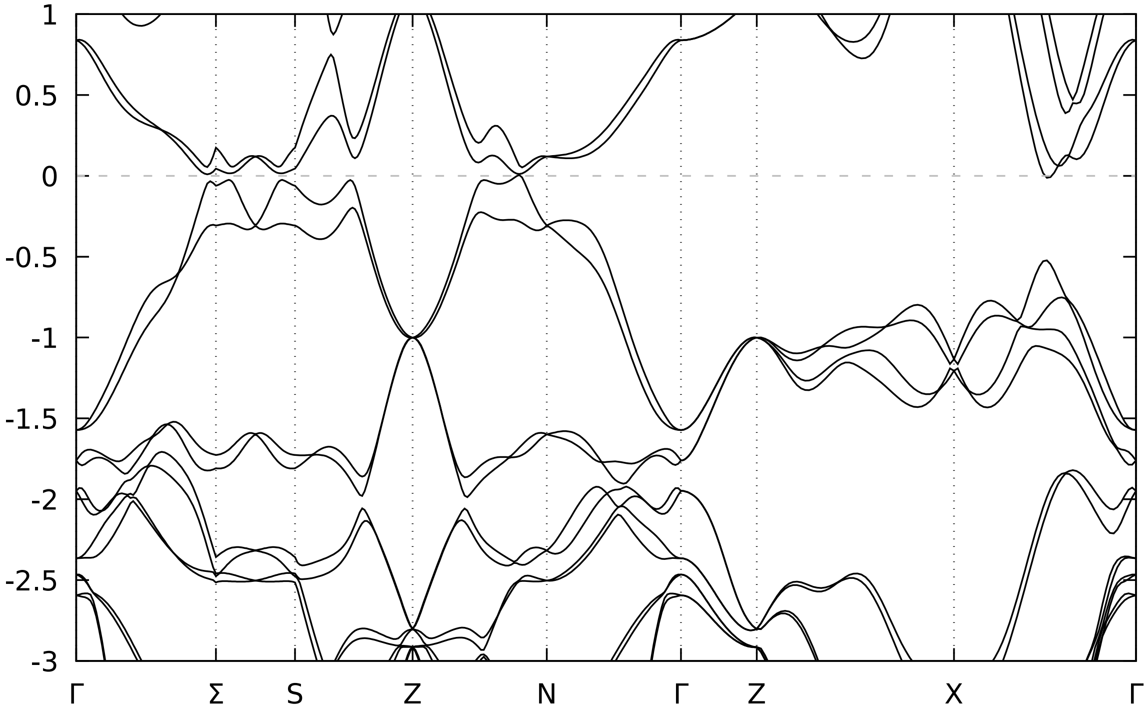

Supplementary Fig. 1: **Calculated band structure of TaAs along high-symmetry lines.**

I. SUPPLEMENTARY NOTE 1

A. Bulk band structure

Figure S1 shows the calculated bulk band structure of TaAs for which we find good agreement with previous works [1], providing a starting point for more detailed analysis discussed in the main text. Figure S2 shows an extended $h\nu$-dependent ARPES data set (cf. Fig. 1 of the main text). We find good agreement with the calculated band dispersion over a wide range of photon energies from $h\nu = 420$ eV to $h\nu = 700$ eV for an inner potential of $V_0 = 25$ eV.

* These authors contributed equally to the present work.; hendrik.bentmann@physik.uni-wuerzburg.de

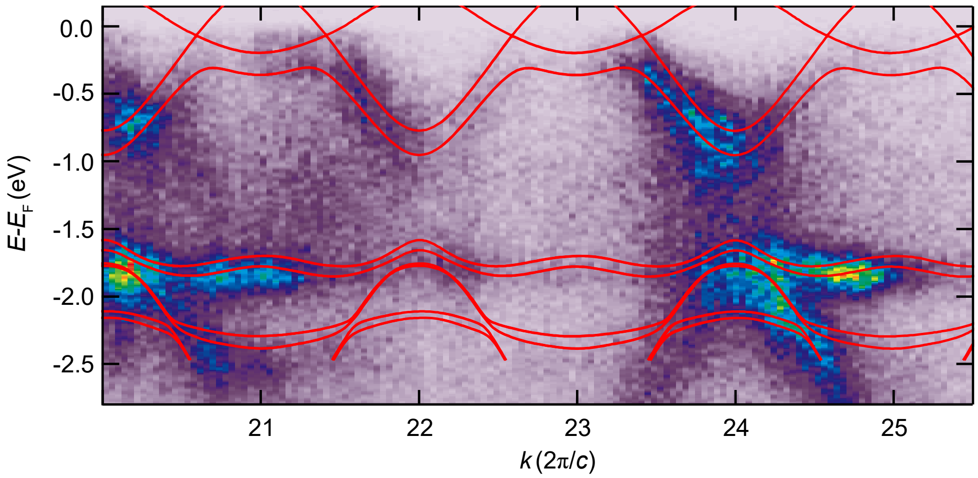

Supplementary Fig. 2: **Band structure along k_z.** $h\nu$-dependent ARPES data set along k_z at $k_x = -0.53$ Å^{-1}, corresponding to the calculated k_x position of the W_2 Weyl nodes (cf. Fig. 2e-f of the main manuscript). The data set was obtained from a photon-energy series from $h\nu = 420$ eV to $h\nu = 700$ eV. The calculated band structure is overlaid on the experimental data.

II. SUPPLEMENTARY NOTE 2

A. Influence of inversion-symmetry breaking on orbital angular momentum

To show explicitly that the formation of orbital angular momentum (OAM) is a direct consequence of inversion-symmetry breaking (ISB) we performed first-principles calculations for the actual crystal structure of TaAs (space group $I4_1md$) with ISB and for a crystal structure corresponding to its supergroup unit cell (space group $I4_1/amd$) without ISB. As can be seen from Fig. S3, OAM vanishes in the absence of ISB and forms in the presence of ISB already in the absence of SOC.

As known from the context of surface states [2, 3], the interplay of ISB and SOC may unfold in two different limits depending on whether ISB or SOC constitutes the dominating energy scale. In a SOC-dominated limit, SOC forms states of total angular momentum J upon which ISB acts as perturbation leading to a splitting into states of opposite SAM. In this case, one expects an opposite OAM of the two spin-split states [2, 3]. In an ISB-dominated scenario, on the other hand, the OAM L remains approximately a good quantum number in the presence of SOC, which acts as a small perturbation compared to ISB. SOC splits the states of given L further into two states of the same L but opposite S, so that the two spin-split branches carry parallel OAM. This is the

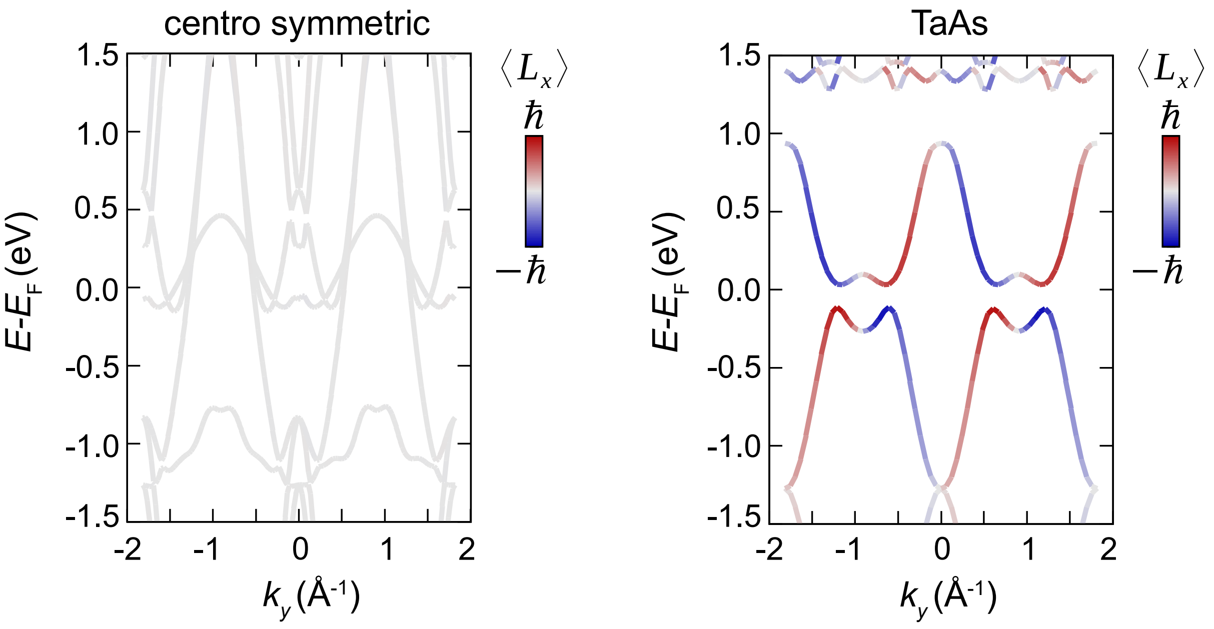

Supplementary Fig. 3: **Artificial tuning of inversion symmetry-breaking (ISB) in TaAs.** Dependence of the band structure and the OAM on the ISB along $k_x = 0$. The crystal structure underlying the calculations were obtained (right) for the non-centrosymmetric TaAs crystal unit cell (space group $I4_1md$) and (left) its supergroup unit cell (space group $I4_1/amd$) in the absence of SOC.

trend we observe here for the bulk bands in TaAs based on our CD measurements and calculations (Figs. 2 and 4 of the main text), revealing an ISB-dominated nature of the spin splitting of the bulk band structure.

This knowledge about the energy hierarchy of ISB and SOC allows for an assignment of each band to a specific OAM manifold. We found that indeed, the conduction band (CB) $c_\pm$ and the valence band (VB) $v_\pm$ in TaAs feature opposite OAM, as can be seen from Figs. 2 and 4 in the main text and as schematically sketched in Fig. S4. The Weyl node, i.e the crossing between VB and CB, is accordingly an intersection of bands carrying different sign of L (Fig. S4). We show this explicitly for a k_x-path in Fig. 4 in the main text for an OAM quantization axis $\langle L_x \rangle$, which aligns parallel to the electron momentum k_x.

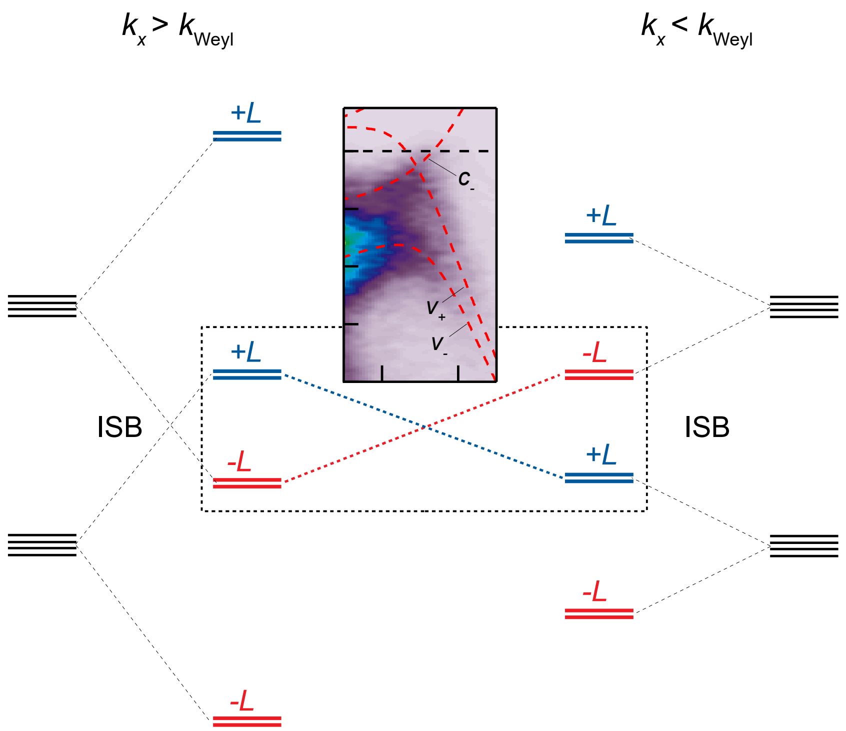

Supplementary Fig. 4: **OAM-dependent band inversion of CB and VB in an ISB-dominated limit.** A case is considered in which ISB constitutes the dominating energy scale [3]. Dominant ISB leads to the formation of states of opposite OAM L. Depending on momentum k, two different types of band-ordering can be found: For $k_x < k_{\mathrm{Weyl}}$ the $-L$-states (red) form the conduction band and lie above the valence band states, which, in turn, is composed of the $+L$ manifold (blue). Passing the Weyl point ($k_x > k_{\mathrm{Weyl}}$) VB and CB intersect, leading to an inverted band-ordering, with the $+L$-states being now energetically above the $-L$ bands. The ARPES data and DFT calculations (Fig. 4 in the main text) show that the Weyl cone in TaAs constitutes a crossing point between bands of opposite OAM.

B. Circular dichroism and orbital angular momentum

Circular dichroism in angle-resolved photoemission spectroscopy (CD-ARPES) has been established as a qualitative probe of orbital angular momentum (OAM) in band structures [2–5]. Using circularly polarized light incident in the xz-plane the CD in the photoelectron angular distribution is defined as:

$$CD(k_x, k_y, E) = I_R(k_x, k_y, E) - I_L(k_x, k_y, E). \tag{1}$$

I_R and I_L are the intensities measured with right and left circularly polarized light. In the present case the xz-plane is a crystalline mirror plane so that the CD is caused solely by the mirror-symmetry breaking of the light electric field vector, given by $\mathbf{E} \approx (0, \pm\mathcal{E}_y, i\mathcal{E}_z)$ for the case of grazing light incidence (see Fig. 2a of the main text). For this situation the CD can also be written as

$$CD(k_x, k_y, E) = I_R(k_x, k_y, E) - I_R(k_x, -k_y, E). \tag{2}$$

One finds $I_R(k_x, \pm k_y, E) \propto |\pm T_y + iT_z|^2$ and for the CD one obtains

$$CD(k_x, k_y) \propto \Im(T_\mathrm{y}^* T_\mathrm{z}), \tag{3}$$

where $T_z = \langle\, \Phi_f\, |\mathcal{E}_z p_z|\, \psi_i \,\rangle$ and $T_y = \langle\, \Phi_f\, |\mathcal{E}_y p_y|\, \psi_i \,\rangle$ are the photoemission matrix elements of the electric field components $\mathcal{E}_z$ and $\mathcal{E}_y$ (here given in the dipole approximation) with the photoelectron final state Φ_f and the initial state ψ_i. Eq. 5 implies that both T_z and T_y must be of appreciable magnitude to generate a sizable dichroism and that k-dependent changes of T_z and T_y directly manifest in a k-dependence of the dichroism.

In Fig. 3 of the main manuscript we show the momentum distribution of the CD, which is reproduced in Fig. S5 for convenience. The CD shows marked changes, even sign inversions, close to the Weyl points W_2. This implies that the matrix elements T_z and/or T_y markedly change at these in-plane momenta. A priori these changes could arise either from a change of the final state wave function Φ_f or of the initial state wave function ψ_i. However, there is obviously no reason to expect a pronounced change of Φ_f, i.e. for states ca. 585 eV above E_F, at these particular momenta. Thus, the measured CD in Fig. 3 implies a strong change of ψ_i near W_2, which is in good agreement with our calculations that show a change in orbital character at the Weyl points as a result of the band inversion between the bands $v_\pm$ and $c_\pm$ (see Fig. 3 of the main text).

Upon further approximation of the final state of the photoelectron as a free-electron-like state, a direct relation between CD and the OAM of the initial state ψ_i can be derived, see Refs. [2, 4, 5]. In the present case, the final state lies ca. 585 eV above the Fermi energy, such that a free-electron-like behavior can be expected [6]. The close agreement between measured CD and calculated OAM for the bulk states in TaAs indicates CD-SX-ARPES as a powerful approach to study OAM-structures in the bulk electronic structures of solids. Although in the present experiments the OAM of the initial state is the most important source determining the momentum dependence of the CD, there might be other cases where the behavior becomes more complex. For example, other

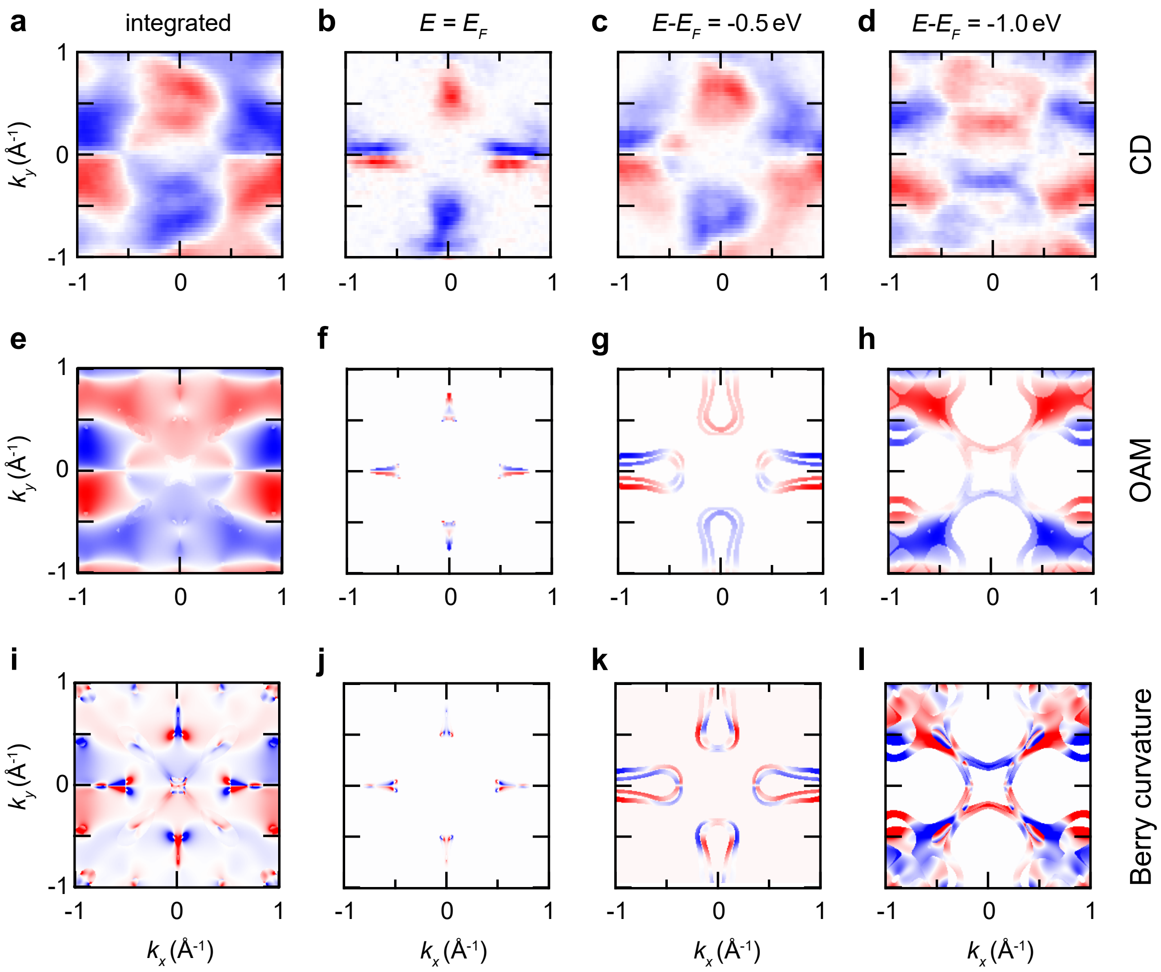

Supplementary Fig. 5: **Systematics of circular dichroism (CD), orbital angular momentum (OAM) and Berry curvature.** (top row) CD-ARPES data set at $h\nu = 590$ eV (cf. Fig. S6). (middle row) Calculated L_x component of the orbital angular momentum (OAM) and (bottom row) calculated Ω_x component of the Berry curvature at $k_z = -0.592\frac{2\pi}{c}$, corresponding to the W_2 Weyl nodes. For CD, OAM and Berry curvature we show momentum distributions at selected binding energies and (left column) integrated over an energy range from $0\,\text{eV}$ to $-1.2\,\text{eV}$.

momentum-dependent changes of the initial-state wave function, such as their depth localization, could give rise to additional momentum dependent effects in the CD signal.

For a more quantitative comparison it is useful to consider the normalized CD:

$$CD_n(k_x, k_y, E) = \frac{I_R(k_x, k_y, E) - I_L(k_x, k_y, E)}{I_R(k_x, k_y, E) + I_L(k_x, k_y, E)}. \qquad (4)$$

Both, CD and CD_n show qualitatively the same behaviour in terms of strong changes in the dichroism near the Weyl points.

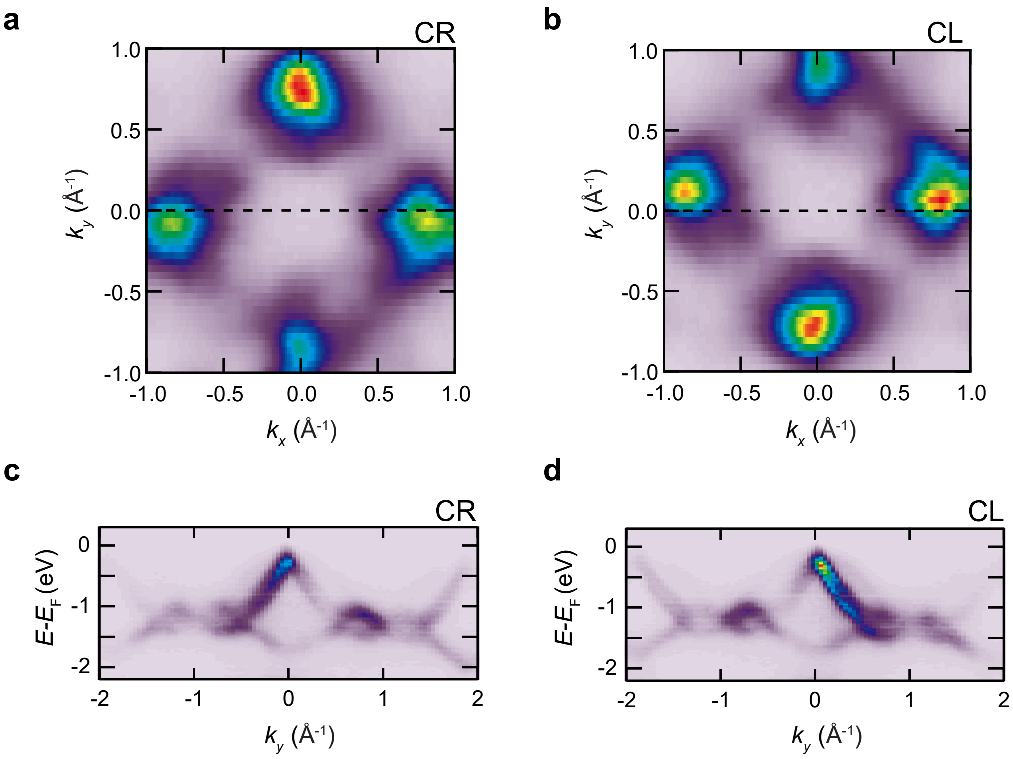

Supplementary Fig. 6: **Circular dichroism.** ARPES data sets of TaAs(001) obtained with left (CL) and right (CR) circularly polarized light at $h\nu\ = 590$ eV incident in the xz plane (cf. Fig. 2a of the main text). The data sets in **a** and **b** correspond to the circular-dichroism momentum distribution shown in Fig. 4a of the main manuscript. The data sets in **c** and **d** correspond to the CD-ARPES band structures shown in Fig. 3a of the main manuscript.

Fig. S6 shows ARPES data sets obtained with right and left circularly polarized light, corresponding to the CD data sets in Fig. 2 and Fig. 3 of the main manuscript. Strong intensity asymmetries relative to the xz plane are directly apparent in the raw data.

C. Spin-resolved ARPES

The spin-resolved ARPES measurements were performed in the experimental geometry shown in Fig. 1b of the main text. Spin-resolved energy distribution curves (EDC) were measured at finite emission angles using the deflection mode of the spectrometer. Figs. S7a,c show spin-resolved EDC with a spin quantization axis along x at four different locations in momentum space, as indicated in Fig. S7b. The relative sign of the measured spin polarization at the four locations

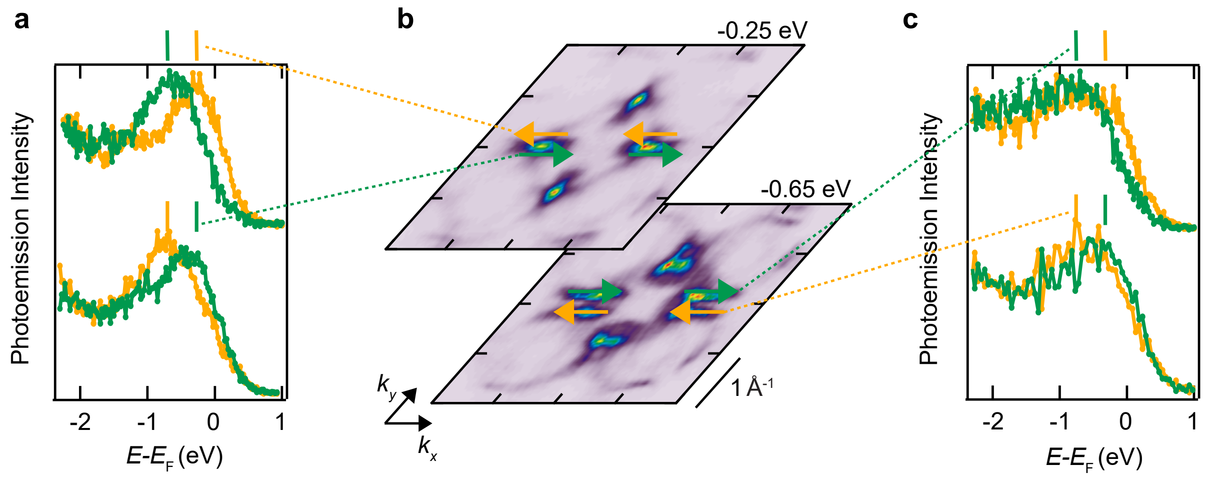

Supplementary Fig. 7: **Spin polarization. a** Spin-resolved energy distribution curves (EDC) taken at $k_x = -1\,\text{Å}^{-1}$ and $h\nu = 590\,\text{eV}$. **c** Spin-resolved EDC taken at $k_x = +1\,\text{Å}^{-1}$. In both cases EDC at $\pm k_y$ are shown, as indicated in the ARPES momentum distributions in **b**. The spin quantization axis is along x. Data sets at $+k_y$ ($-k_y$) were measured with left (right) circularly polarized light.

reflects the crystalline symmetries. In particular, the spin polarization S_x reverses for $k_y \to -k_y$ and remains unchanged for $k_x \to -k_x$.

The measurements were performed using circularly polarized light incident in the xz plane (Fig. 1b of the main text). Data sets at positive (negative) k_y were taken using left (right) circular polarization CL (CR). In this case, the experimental geometries of measurements at positive k_y and negative k_y are related by mirror symmetry, because mirror reflection imposes $k_y \to -k_y$ and $CL \to CR$. In this way the experiments respect the crystalline mirror symmetry of TaAs with respect to the xz plane. As a result, the polarized light excites equivalent orbitals of the initial-state wave functions $\psi_{i,\pm k_y}$ at positive and negative k_y, which is reflected by the relationship $I_R(k_x, k_y, E) = I_L(k_x, -k_y, E)$ for the photoemission intensities that is nicely comfirmed by the experimental data in Fig. S6. In this regard, the sign change of the photoelectron spin polarization for $k_y \to -k_y$ (Figs. S7a-c) reflects the corresponding sign change in S_x of ψ_i, that is seen in our calculations (Figs. S7e-f) and that is imposed by the crystalline mirror symmetry.

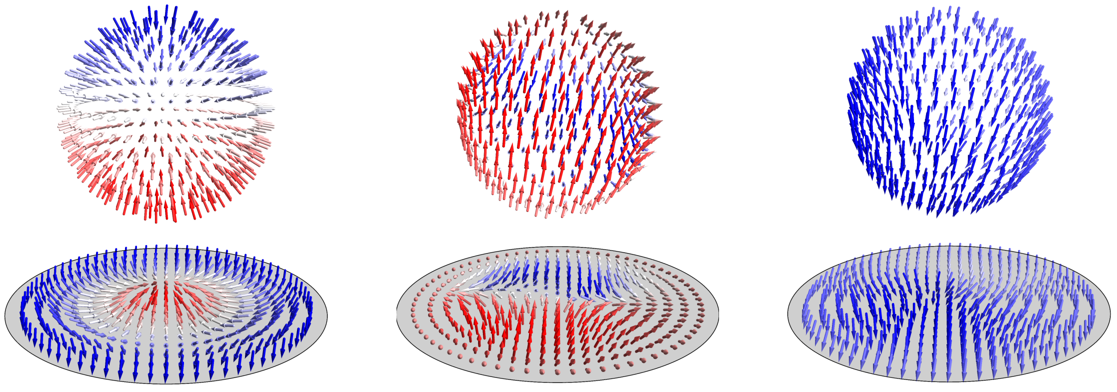

Supplementary Fig. 8: **Momentum texture topology.** Berry curvature, OAM and SAM calculated on a small sphere around a W_2 Weyl node and the corresponding azimuthal equidistant projection. The momentum textures of Berry curvature and OAM carry a non-trivial Pontryagin index of $S = 1$, reflecting the non-trivial topology of the Berry flux monopole. The momentum texture of the SAM carries a trivial Pontryagin index of $S = 0$.

III. SUPPLEMENTARY NOTE 3

A. Topology of OAM, SAM and Berry curvature textures

To analyze the momentum texture topology of the observables (OAM, SAM and BC) around the W_2 point [0.509, 0.037, -0.316] Å^{-1}. We sample the surface of the sphere with radius $r = 0.03$ Å^{-1} centered around the Weyl point on a 50×50 (ϕ, θ) grid (ϕ: polar angle, θ: azimuthal angle) to calculate the related expectation values by diagonalizing a Wannier Hamiltonian. The winding numbers were obtained by performing a stereographic projection onto a plane followed by the calculation of the Pontryagin index in the plane. Our analysis yields a non-trivial winding of the BC and OAM, and a trivial winding of the SAM (as shown in Fig. S8). Thus, the non-trivial topology of the Weyl bands is encoded in their orbital degrees of freedom.

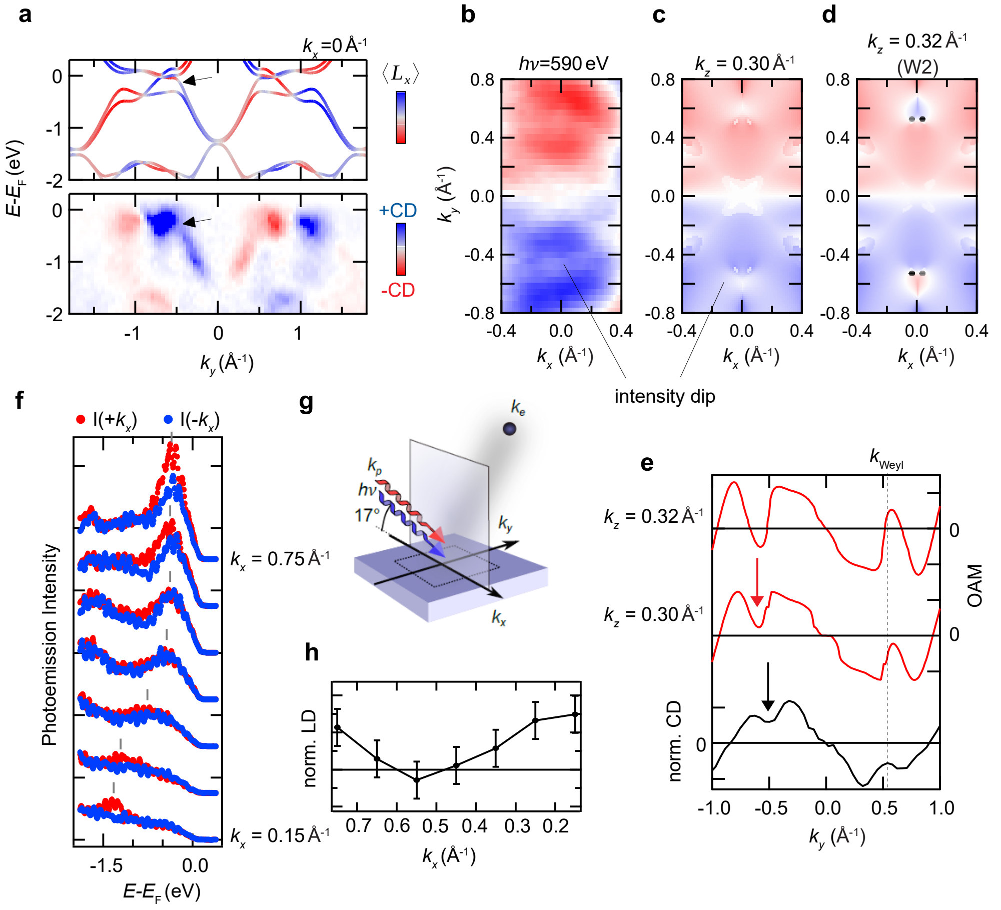

Supplementary Fig. 9: **Supplementary dichroism data and OAM calculations at W2 Weyl nodes.** **a**, Upper panel: calculated bulk band dispersion of TaAs projected on the component L_x of the orbital angular momentum (OAM). The cut along k_y is taken at $k_z = -0.59\frac{2\pi}{c}$ and $k_x = 0\,\text{Å}^{-1}$. Bottom panel: the calculation is compared to a corresponding CD-ARPES data set in the bottom panel, obtained at $h\nu = 590$ eV. The red/blue color code represents the circular dichroism (CD) measured with circularly polarized X-rays incident in the xz-plane (see panel **g**). **b-d**, CD-ARPES data and calculated L_x at k_z = -0.3 Å^{-1} and -0.32 Å^{-1} obtained by integrating over an energy range from $0\,\text{eV}$ to $-1.2\,\text{eV}$, the width of the bands $v_{\pm}$. **e**, Momentum distribution curves at $k_x = 0\,\text{Å}^{-1}$ of the CD and the OAM corresponding to the panels in **b-d**. **f**, Energy distribution curves along k_x. Intensity differences between $I(k_x)$ and $I(-k_x)$ reflect the linear dichroism (LD) [see text for details]. **g**, Sketch of the experimental geometry. **h**, LD evaluated from the data in **f**.

IV. SUPPLEMENTARY NOTE 4

A. Supplementary dichroism data at W_2 Weyl nodes

In Fig. 3 of the main text we discuss signatures of the Berry flux monopoles at the W_2 Weyl nodes as manifested in the L_x component of the OAM. Our calculations further show that also the L_y component shows a characteristic modulation near the W_2 nodes. Due to the fourfold rotational symmetry of the band structure about the [001] axis, the behavior of L_y at the W_2 nodes near the k_x axis is equivalent to the behavior of L_x at the W_2 nodes near the k_y axis and can thus be addressed in the same experimental geometry, as presented in Fig. S9.

Focusing first on our calculations in Figs. S9a,c-d, we see that the L_x component along k_y (near $k_x = 0$) shows a modulation near the W_2 nodes. This is best seen in Figs. S9d where the sign of L_x reverses in small $k_x - k_y$ region near the W_2 nodes. Compared to the modulation of L_x along k_x (Fig. 3 in the main manuscript) the present effect along k_y is considerably more subtle for two reasons: (i) along the k_z direction the sign reversal occurs only in a small momentum interval Δk_z of ca. 0.02 Å^{-1}, as seen by comparing the calculations at slightly different k_z in Figs. S9c,d; (ii) additionally, along the k_y axis ($k_x = 0$) the energy separation between bands of opposite L_x is small, as seen in Fig. S9a.

Both aspects make it more difficult to address the effect experimentally. In particular, the photoemission process features an inherent k_z-broadening δk_z, which is due the exponential damping of the final-state wave function and unrelated to instrumental uncertainties [6]. Estimating a characteristic decay length λ of the final state of ∼2 nm, results in $\delta k_z = \lambda^{-1}$ ∼0.05Å^{-1}, which is considerably larger than Δk_z. In accordance with this estimation, our CD experiments do not resolve a full sign reversal of L_x along the k_y axis. Nevertheless, our data in Figs. S9a,b,e reveal a dip-feature in the CD signal at approximately the k_y-position of the W_2 nodes. This is most clearly seen in the MDC curves in Fig. S9e, where the normalized CD signal shows a dip-feature near the W_2 nodes that is comparable to the k_y-dependence of the calculated L_x. We attribute the fact that the dip is less pronounced in the experimental data than in the calculation mainly to the aforementioned intrinsic broadening δk_z. Nevertheless, the good qualitative agreement of the experimental data and calculations in Fig. S9e provides evidence that, in addition to the OAM component discussed in Fig. 3 of the main manuscript, also the second in-plane component of the OAM is modulated in the vicinity of the W_2 nodes.

Further experimental evidence for changes in the orbital character near the W_2 nodes is provided by linear-dichroism (LD) data in Fig. S9f. For light incident in the xz-plane (Fig. 3g) the LD in the photoelectron angular distribution is defined as: $LD(k_x, k_y, E) = I(k_x, k_y, E) - I(-k_x, k_y, E)$. The LD represents the intensity asymmetry relative to the yz-plane. Similarly as for the CD, one finds $LD(k_x, k_y) \propto \Re(T_{\mathrm{x}}^* T_{\mathrm{z}})$, where $T_z = \langle \Phi_f | \mathcal{E}_z p_z | \psi_i \rangle$ and $T_x = \langle \Phi_f | \mathcal{E}_x p_x | \psi_i \rangle$ are the photoemission matrix elements of the electric field components $\mathcal{E}_z$ and $\mathcal{E}_x$. Its similar origin as the CD, implies that the LD is also sensitive to the orbital composition of the initial state. We have previously employed the LD to experimentally establish the orbital texture of the Fermi-arc surface states in the Weyl semimetal TaP(001) [7]. Fig. S9f shows energy distribution curves obtained along positive and negative k_x. The corresponding LD is plotted in Fig. S9h. Close to the W_2 nodes, i.e. near $k_x =$ 0.5-0.6 Å^{-1}, the LD exhibits a minimum. Thus, in agreement with our CD data in Fig. 3 and Fig. S9, the LD data indicates a modulation of the orbital character across the W_2 nodes.

B. Curvature analysis of ARPES data near the Weyl cone

In order to analyze the ARPES data near the W_2 Weyl nodes in more detail, we carried out a curvature analysis [8] of the 2D data sets shown in Fig. 4d-e in the main manuscript. The results for left and right circular polarized light are shown in Fig. 10. The features in the 2D curvature data sets in in Fig. 10a-b resemble those in the respective 2D intensity data sets in Fig. 4d-e and also agree well with the overlaid calculated band dispersion. However, one can see that in the curvature data set for I_L the band v_+/c_- can be traced to higher energies. In the intensity data sets in Fig. 4d-e this feature becomes less apparent towards E_F due to a drop in spectral weight. While the curvature analysis allows one to trace the band v_+ reasonably well up to the Weyl point, additional signatures of the band c_- complicate a clear assignment above the Weyl point. The curvature analysis therefore further supports that the red/upwards dispersing and blue/downwards dispersing branches of v_+/c_-, which cross at the Weyl point, are predominantly excited by left and right circularly polarized light and thus carry opposite OAM, in agreement with the calculations.

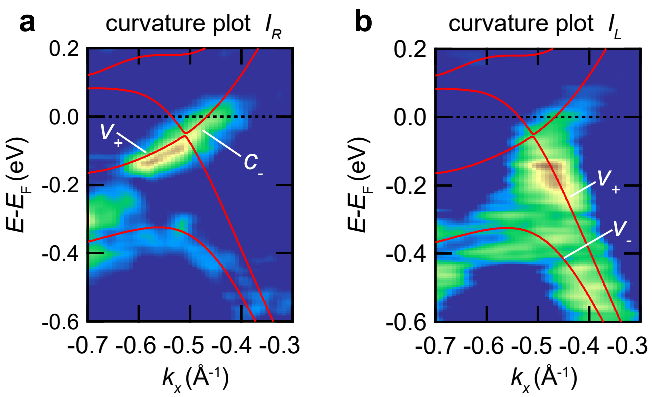

Supplementary Fig. 10: **Curvature analysis of ARPES data near the Weyl cone. a,b** Two-dimensional curvature analysis of the ARPES data sets shown in Fig. 4d,e in the main manuscript, taken with right and left circularly polarized light (I_R and I_L). Red lines correspond to the DFT-calculated band dispersions.

C. EDC model near the Weyl-cone with different light polarizations

In order to rationalize deviations of the observed circular dichroism and the calculated OAM close on a small energy scale around the Weyl node (Fig. 4b), we modeled energy distribution curves (EDC) for intensities and CD. The EDC were modeled at different momenta based on the DFT-calculated band positions and the mean experimentally observed linewidth broadening of $\mathrm{FWHM} = 200\,\mathrm{meV}$. Besides the energy resolution ($50\,\mathrm{meV}$), the EDC linewidths are affected by an intrinsic broadening along momentum k_z due to exponential damping of the final-state wave function into the bulk, finite angular resolution ($\sim$0.1° corresponding to $\sim$0.02 Å^{-1}), and intrinsic broadening, e.g. due to defects. The results are shown in Fig. **??** and compared to the measured EDC and CD (right panel).

The simulated EDC were estimated assuming [case (i), left panels] a constant, momentum-independent intensity and [case (ii), middle panels] a momentum-dependent reduction of intensity for the downward dispersing branch of the Weyl cone towards higher energies, which is observed in Fig. 4e in the main part.

Each spectrum is modeled by a sum of three Gaussian profiles multiplied by a Fermi-Dirac distribution. Positive, negative or zero OAM in the initial state, as obtained by DFT, are assumed to be selectively probed by right (blue), left (red) or both circular light polarizations, respectively. For case (i) we assumed fully selective excitation (i.e. $100\,\%$ CD for an OAM-polarized bands) and for

case (ii) we assumed a CD of 80 $\%$ for OAM-polarized bands, as estimated from the experimental data and in agreement with previous works on topological surface states [9].

One can see that, left of the WP (top row in Fig. **??**), the CD sign reversal above the WP is almost entirely suppressed already for the ideal scenario (i) and is fully suppressed for the case (ii). A comparably clear sign reversal is, however, visible right of the WP for both scenarios (bottom row in Fig. **??**), in agreement with the experimental data. The missing sign reversal left of the WP is thus attributed to the smaller energy distance of the two bands v_+ and c_-, the experimental broadening and the momentum-dependent intensity variation. For the case (ii) there is also a finite CD signal at the Weyl point (middle row in Fig. **??**), originating from different intensities for the upwards and downwards dispersing parts of the band v_+. For an additional visualization of the CD behavior explained above a two-dimensional color plot of the simulated CD is shown in Fig. S12 (a) and (b) for the scenarios (i) and (ii), respectively.

V. SUPPLEMENTARY NOTE 5

A. LaAlGe

1. Computational Details

LaAlGe is investigated by following the approach described in *theoretical details* of the manuscript. The Brillouin zone is sampled on a $8 \times 8 \times 8$ regular mesh with a plain-wave cut-off energy of $300\,\mathrm{eV}$. A Wannier Hamiltonian is created by projecting onto La s, p, d, f Al s, p and Ge s, p orbitals.

B. Bulk band structure

Figure 13 shows the calculated band structure which is in good agreement with reference [10]. The low-energy bands forming the Weyl points have a dominating La $5d$ and $4f$ character.

	W_1	W_2	$W_3{}'$	$W_3{}''$
Type	1	2	1	1
BC	1	1	1	1
OAM	1	0	1	1
SAM	0	0	0	0

Table I: **Weyl point topology of LaAlGe.**

1. Weyl point topology

The Pontryagin index of the Berry curvature, the SAM and OAM of the Weyl points are given in Table I by following the Weyl point nomenclature introduced in reference [10]. As for the TaAs family, we find a non-trivial winding of the OAM at all type-1 Weyl points, but a trivial winding for the type-2 W_2 node. Based on our calculations, the OAM carries a non trivial Pontryagin index at all type-1 Weyl nodes in the considered compounds (TaAs, TaP, LaAlGe) of the non-symmorphic space group I_4md.

2. Type-II Weyl node OAM polarization

Figure 14 shows a cut along k_x through a W_2 point, for which the OAM winds trivially. The point is characterized indeed by a vanishing L_x component and the absence of a sign change. Further, the second conduction and valence bands do not show a sign change in the L_x polarization at the Weyl node (cf. Fig 4a-e of the manuscript).

---

[1] H. Weng, C. Fang, Z. Fang, B. A. Bernevig, and X. Dai, Phys. Rev. X **5**, 011029 (2015).

[2] J.-H. Park, C. H. Kim, J.-W. Rhim, and J. H. Han, Phys. Rev. B **85**, 195401 (2012).

[3] V. Sunko, H. Rosner, P. Kushwaha, S. Khim, F. Mazzola, L. Bawden, O. J. Clark, J. M. Riley, D. Kasinathan, M. W. Haverkort, et al., Nature **549**, 492 (2017).

[4] S. R. Park, J. Han, C. Kim, Y. Y. Koh, C. Kim, H. Lee, H. J. Choi, J. H. Han, K. D. Lee, N. J. Hur, et al., Phys. Rev. Lett. **108**, 046805 (2012).

[5] M. Schüler, U. D. Giovannini, H. Hübener, A. Rubio, M. A. Sentef, and P. Werner, Sci. Adv. **6**, eaay2730 (2020).

[6] V. N. Strocov, Journal of Electron Spectroscopy and Related Phenomena **130**, 65 (2003).

[7] C.-H. Min, H. Bentmann, J. N. Neu, P. Eck, S. Moser, T. Figgemeier, M. Ünzelmann, K. Kissner, P. Lutz, R. J. Koch, et al., Phys. Rev. Lett. **122**, 116402 (2019).

[8] P. Zhang, P. Richard, T. Qian, Y.-M. Xu, X. Dai, and H. Ding, Review of Scientific Instruments **82**, 043712 (2011).

[9] M. R. Scholz, J. Sánchez-Barriga, J. Braun, D. Marchenko, A. Varykhalov, M. Lindroos, Y. J. Wang, H. Lin, A. Bansil, J. Minár, et al., Phys. Rev. Lett. **110**, 216801 (2013).

[10] S.-Y. Xu, N. Alidoust, G. Chang, H. Lu, B. Singh, I. Belopolski, D. S. Sanchez, X. Zhang, G. Bian, H. Zheng, et al., Sci. Adv. **3**, e1603266 (2017).

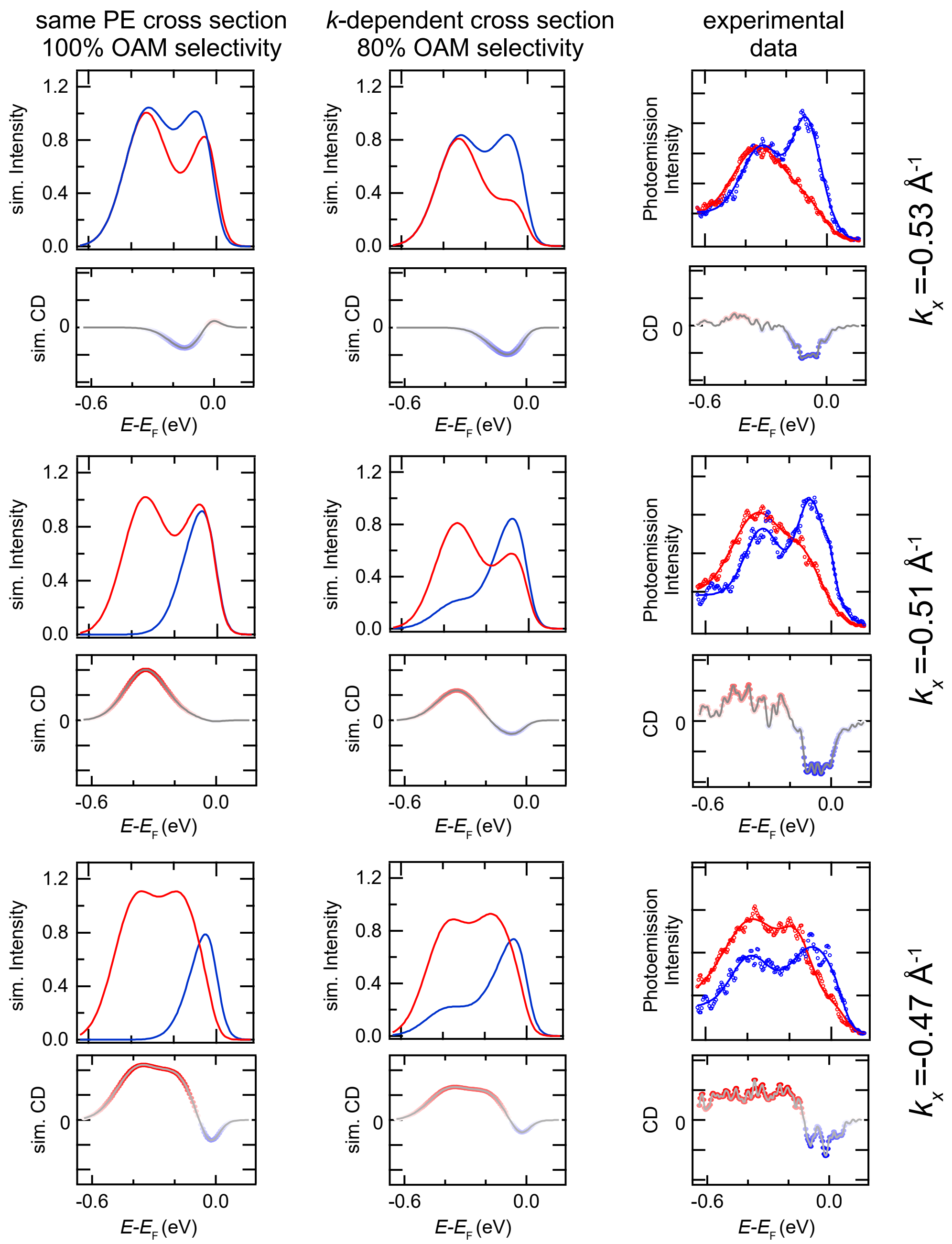

Supplementary Fig. 11: **Energy distribution curves (EDC) and circular dichroism (CD) close to the W_2 Weyl point.** The first two columns show modeled EDC for intensities and CD. The third column shows the corresponding experimental data for different k_x around the Weyl point, as labeled on the right (the calculated Weyl point is at $k_x = 0.51$ Å^{-1}). Blue and red color indicate data taken with right and left circularly polarized light. The model is based on the energy positions of v_-, v_+ and c_- obtained by DFT with a mean Gaussian peak broadening as obtained from the ARPES data (see text). In the first column a k_x-independent cross section and a full OAM selectivity is assumed, while in the second column, cross sections vary for different momenta (see text) and the OAM selectivity was set to $80\,\%$.

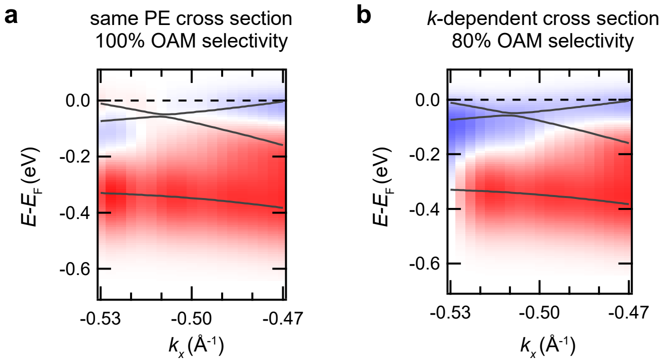

Supplementary Fig. 12: **Model for circular dichroism (CD) close to the W_2 Weyl point**. Two-dimensional color plot of the simulated CD using the same parameters as in Fig. S11 (left and middle column) for **a** and **b**, respectively (see text).

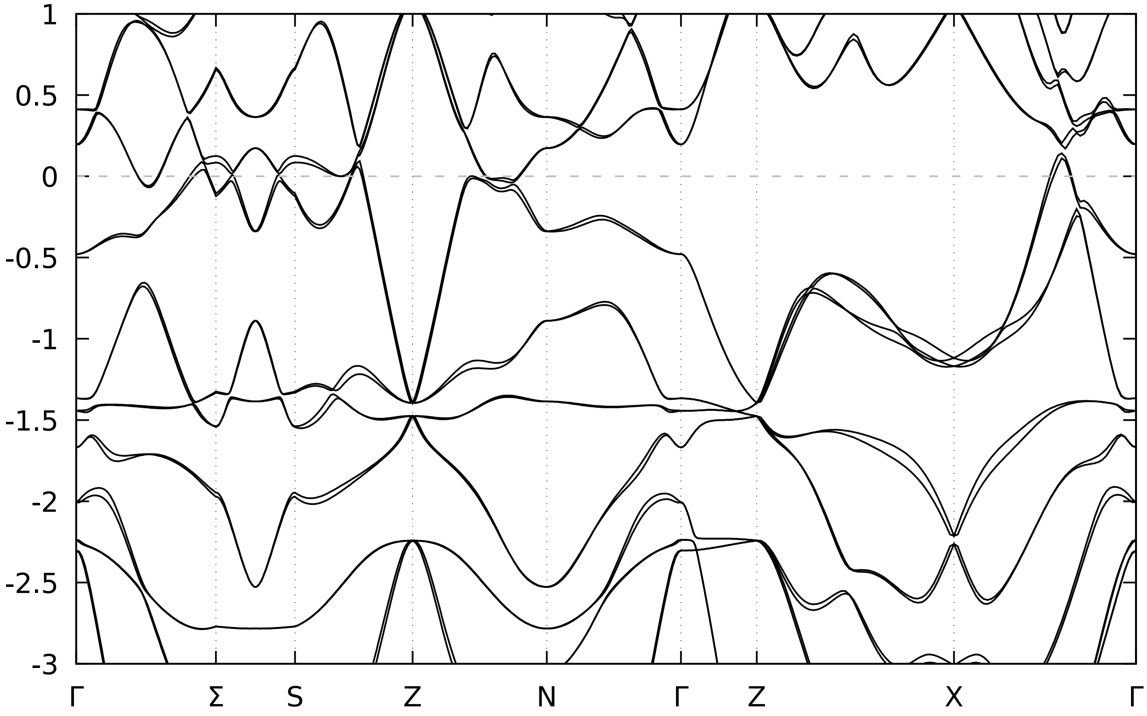

Supplementary Fig. 13: **LaAlGe electronic properties.** Calculated band structure along high-symmetry lines.

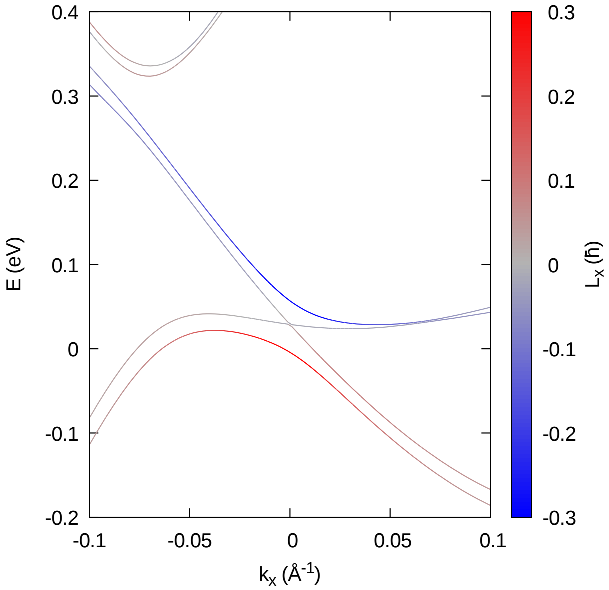

Supplementary Fig. 14: **Band resolved OAM.** L_x polarization along k_x at the W_2 node.
